# Systemic inflammatory indices and age-dependent severity in acute appendicitis: a retrospective cohort study

**DOI:** 10.3389/fimmu.2025.1620459

**Published:** 2025-07-15

**Authors:** Catalin Vladut Ionut Feier, Andrei Motoc, Calin Muntean, Razvan Constantin Vonica, Vasile Gaborean, Sorin Olariu, Marius Sorin Murariu

**Affiliations:** ^1^ Abdominal Surgery and Phlebology Research Center, Victor Babeş University of Medicine and Pharmacy, Timisoara, Romania; ^2^ First Surgery Clinic, “Pius Brinzeu” Clinical Emergency Hospital, Timişoara, Romania; ^3^ Department of Anatomy and Embryology, “Victor Babes” University of Medicine and Pharmacy Timisoara, Timisoara, Romania; ^4^ Medical Informatics and Biostatistics, Department III-Functional Sciences, “Victor Babeş” University of Medicine and Pharmacy Timişoara, Timişoara, Romania; ^5^ Preclinical Department, Discipline of Physiology, Faculty of Medicine, “Lucian Blaga” University of Sibiu, Sibiu, Romania; ^6^ Department of Oncology, Elysee Hospital, Alba Iulia, Romania; ^7^ Thoracic Surgery Research Center, “Victor Babeş” University of Medicine and Pharmacy Timişoara, Timişoara, Romania; ^8^ Department of Surgical Semiology, Faculty of Medicine, “Victor Babeş” University of Medicine and Pharmacy Timişoara, Timişoara, Romania

**Keywords:** acute appendicitis, systemic inflammatory biomarkers, immunosenescence, systemic immune-inflammation index, platelet-to-lymphocyte ratio

## Abstract

**Background:**

Acute appendicitis (AA) remains the most common cause of emergency abdominal surgery, yet achieving precise preoperative risk stratification is still challenging, particularly among elderly patients. Recent interest has focused on systemic inflammatory biomarkers and the role of immunosenescence in influencing disease progression.

**Materials and methods:**

We retrospectively analyzed 407 adult patients who underwent appendectomy over a six-year period at a tertiary hospital. Patients were grouped by age and histopathological subtype. Preoperative blood counts were used to calculate systemic inflammatory indices, including NLR, PLR, SII, SIRI, and AISI. Associations between biomarkers and histopathological severity were assessed using multivariable multinomial logistic regression, with adjustments for age and hospitalization duration.

**Results:**

Higher neutrophil counts and elevated PLR were significantly associated with gangrenous appendicitis (p < 0.001 and p = 0.047, respectively). Increased SIRI and neutrophil levels predicted phlegmonous appendicitis (p = 0.020 and p < 0.001). Age independently correlated with more severe histopathological forms. Distinct variations in inflammatory profiles were observed across different age groups and histological categories.

**Conclusion:**

Systemic inflammatory indices, particularly NLR, PLR, SII, and SIRI, hold considerable promise for enhancing preoperative stratification in acute appendicitis. Their integration into clinical practice could improve diagnostic accuracy, especially in older patients affected by immunosenescence.

## 1 Introduction

Acute appendicitis (AA) is a leading cause of emergency abdominal surgery worldwide, with an estimated lifetime risk of 7% and a perforation rate reaching 20% in delayed cases (1, 2). While its pathophysiology traditionally involves luminal obstruction, bacterial invasion, and progressive mucosal necrosis, growing evidence highlights a central role for dysregulated immune responses in modulating disease severity (3, 4). The timely and accurate differentiation between uncomplicated and complicated appendicitis remains a diagnostic challenge—particularly in vulnerable populations such as the elderly—where clinical scoring systems and imaging modalities often lack optimal specificity and accessibility (5).

The last decade has seen a paradigm shift toward immunologically informed diagnostics, with hematologic indices derived from complete blood counts offering an appealing, low-cost surrogate for systemic immune activation. Ratios such as neutrophil-to-lymphocyte (NLR) and platelet-to-lymphocyte (PLR) have gained traction as predictors of complicated appendicitis, albeit with heterogeneous results across populations (6, 7). Beyond their statistical associations, elevated NLR is mechanistically linked to neutrophil extracellular trap (NET) release and NLRP3 inflammasome activation—processes that exacerbate epithelial injury and peritoneal inflammation (8). Likewise, PLR may reflect a hypercoagulable microenvironment, potentiating thromboinflammatory cascades involved in gangrenous progression (6, 9).

To improve diagnostic precision, composite indices such as the systemic immune-inflammation index (SII) and systemic inflammation response index (SIRI) incorporate neutrophils, monocytes, lymphocytes, and platelets into multidimensional immune signatures (10, 11). These indices offer insights not only into overall inflammatory burden but also into the balance between innate and adaptive immunity. However, their clinical adoption remains limited, in part due to insufficient validation across different age groups and histopathological subtypes. Notably, in geriatric patients, the combined use of SII, PLR, and AISI may provide additive value in predicting severe histological forms of appendicitis (10).

Moreover, aging-related immune remodeling—commonly referred to as immunosenescence—has been proposed in the literature as one of several mechanisms potentially influencing leukocyte dynamics in elderly patients. While our study was not designed to explore this mechanism directly, we considered it a relevant context for interpreting age-related biomarker variation. Few studies, however, have accounted for age-related immunologic variance in their biomarker analyses (12). A recent systematic review and meta-analysis has highlighted the potential utility of the systemic immune-inflammation index (SII) as a biomarker for appendicitis, demonstrating robust performance in differentiating between appendicitis and control groups, as well as between complicated and non-complicated cases across both adult and pediatric populations (13).

In this context, we aim to assess the diagnostic relevance of systemic inflammatory indices—including NLR, PLR, SII, SIRI, and AISI—as accessible hematologic markers potentially influenced by age-related factors. While no formal immunologic profiling was performed, the study explores how these indices vary across age groups and histopathological forms.

## 2 Materials and methods

For this retrospective study, patient records were reviewed for individuals who underwent appendectomy at the First Surgery Clinic of the Pius Brînzeu County Emergency Clinical Hospital in Timişoara, Romania, a tertiary care center. Data were collected over a six-year period, from January 1, 2019, to October 31, 2024.

Accordingly, several inclusion criteria were established for the development of this study. Data were considered for patients who underwent surgical intervention for the treatment of acute appendicitis during the specified period. Only patients aged over 18 years were included. Furthermore, given the known impact of neoplastic pathology on the inflammatory status (14, 15), we restricted the cohort to patients without active malignancies, a history of malignant disease, or any prior oncologic treatments such as chemotherapy, radiotherapy, or immunotherapy.

Due to the extended study period, which encompassed the COVID-19 pandemic, additional inclusion criteria were necessary. The global impact of the pandemic on surgical practices is well-documented, and infection with the novel coronavirus posed significant challenges to healthcare systems worldwide. Moreover, it is widely recognized that COVID-19 infection substantially alters the inflammatory status of patients (16–18). Therefore, only patients without a documented history of COVID-19 infection and who did not contract the virus during hospitalization were included in the study. During the study period overlapping with the COVID-19 pandemic, all patients admitted to the surgical clinic were subjected to a standardized screening protocol. Upon admission, each patient underwent RT-PCR testing for SARS-CoV-2 and was accommodated in designated isolation rooms for 24 hours, pending the result. For those requiring emergency surgery, testing was performed immediately upon presentation, and patients were placed in designated postoperative isolation rooms while awaiting test results. Patients who tested positive were transferred to specialized COVID-19 treatment units. Consequently, no patients with confirmed or suspected COVID-19 infection were included in the study cohort. This institutional protocol was strictly applied throughout the pandemic period, thereby minimizing the risk of including asymptomatic carriers and preserving the reliability of systemic inflammatory marker assessment.

After meeting the inclusion criteria, data were collected for comprehensive statistical analysis and interpretation.

Given the variability of the inflammatory status across different ages, patients were stratified into three age groups:

- Young adults aged 18–35 years (G1),
- Adults aged 36–55 years (G2),
- Older adults aged over 56 years (G3).

The cutoff of 56 years was selected based on literature indicating that immunosenescence and age-related systemic inflammation typically begin in the sixth decade of life, marking a biologically relevant shift in immune function (19, 20).

Demographic data (gender, age, and urban/rural residence) were collected.

The preoperative blood count parameters taken into consideration were as follows:

- Lymphocyte (Lym);
- Monocyte (Mon);
- Neutrophil (Neu);
- Platelet (Pla).

Various inflammatory ratios were calculated, including the following:

- NLR (neutrophil/lymphocyte ratio) = Neu/Lym;
- LMR (lymphocyte/monocyte ratio) = Lym/Mon;
- PLR (platelet/lymphocyte ratio) = Pla/Lym;
- AISI (Aggregate Index of Systemic Inflammation) = (Neu × Mon × Pla)/Lym;
- SIRI (Systemic Inflammation Response Index) = (Mon × Neu)/Lym;
- SII (Systemic Immune-Inflammation Index) = (Neu × Pla)/Lym.

In addition to these parameters, the presence of symptoms at admission (nausea, vomiting) and clinical signs (fever, peritoneal irritation, abdominal guarding) was assessed. Furthermore, the length of hospital stay and the type of surgical intervention (laparoscopic vs. open appendectomy) were analyzed.

The histopathological examination of the surgical specimens was categorized into three groups:

- Catarrhal appendicitis (HP1),
- Phlegmonous appendicitis (HP2),
- Gangrenous appendicitis (HP3).

Perforated appendicitis was not classified as a separate entity; cases presenting histological features of transmural necrosis, including those with gross or microscopic perforation, were included in the gangrenous appendicitis (HP3) category.

Postoperative complications were also evaluated, focusing on the incidence of surgical site infection. Additionally, postoperative mortality and the presence or absence of generalized peritonitis were recorded.

Initially, a total of 423 adult patients who underwent appendectomy (either open or laparoscopic) were identified. Among them, 3 patients had histopathological findings consistent with mucinous neoplasm, 5 had a documented history of oncologic disease with prior chemotherapy, and in 8 cases, essential clinical data were incomplete or missing. Following the application of the predefined inclusion and exclusion criteria, the final study cohort consisted of 407 eligible patients.

The study was conducted in compliance with the principles outlined in the Declaration of Helsinki and received approval from the Ethics Committee of the “Pius Brînzeu” Emergency County Clinical Hospital in Timişoara, Romania (Approval No. 497/07 November 2024). In accordance with national regulations and institutional policy, informed consent was waived for retrospective analyses using anonymized patient data.

### 2.1 Statistical analysis

For the statistical analysis and interpretation of results, we utilized IBM SPSS Statistics 25 software for Windows (IBM, Armonk, NY, USA). To determine if numerical data adhered to a normal distribution, the Shapiro-Wilk test was employed with a significance level set at p < 0.05 to indicate normality. Descriptive statistics encompassing measures of central tendency (mean (M)) and dispersion (Standard Deviation (SD)) were applied to summarize numerical variables. For categorical data, frequency distributions and percentages were computed to illustrate variations across different study periods. To compare two independent groups statistically significant differences were assessed using the Student’s t-test. The Mann-Whitney test was used to compare two independent groups when at least one subgroup did not follow a normal distribution, for continuous variables with normal distribution and homogeneous variances (as assessed by Levene’s test), comparisons across multiple groups were performed using one-way ANOVA. For categorical variables, group comparisons were conducted using the Chi-square test or Fisher’s exact test, depending on the expected frequency distribution. Multivariable multinomial logistic regression analysis was performed to assess the independent associations between systemic inflammatory indices (e.g., NLR, PLR, SII, SIRI, AISI) and the histopathological subtypes of acute appendicitis. Odds ratios (ORs) with 95% confidence intervals (CIs) were calculated to evaluate the strength of associations. The model was adjusted for age and other relevant covariates to control for potential confounders. A p-value less than 0.05 was deemed statistically significant across all analyses indicating that observed findings were unlikely due to random variation alone.

## 3 Results

This retrospective study, conducted over a six-year period, included a cohort of 407 patients who underwent appendectomy at the First Clinic of Surgery of the Pius Brînzeu County Emergency Clinical Hospital in Timişoara.

The demographic characteristics of these patients depending of group age are summarized in **Table 1**. Moreover, the symptoms and signs patients presented with at the time of admission, are presented in **Table 2**.

**Table 1:** Demographic characteristics.

Characteristic	All, n=407	G1, n=211	G2, n=127	G3, n=69	p
Age, years (M ± SD)	38.94 ± 16.63	25.83 ± 5.2	45.09 ± 5.91	67.7 ± 7.86	<0.001
Gender, men	243 (59.7%)	127 (60.2%)	80 (63%)	36 (52.2%)	0.33
Urban	216 (53.1%)	113 (53.6%)	64 (50.4%)	39 (56.5%)	0.699

**Table 2:** Signs and symptoms.

Signs and symptoms	All, n=407	G1, n=211	G2, n=127	G3, n=69	p
Symptoms					
Nausea	238 (59.7%)	126 (59.7%)	76 (59.8%)	36 (52.2%)	0.507
Vomiting	149 (36.6%)	81 (38.4%)	45 (35.4%)	23 (33.3%)	0.711
Signs					
Fever Peritoneal irritation Abdominal guarding	133 (32.7%) 57 (14%) 28 (6.9%)	66 (31.3%) 33 (15.6%) 15 (7.1%)	42 (33.1%) 16 (12.6%) 9 (7.1%)	25 (36.2%) 8 (11.6%) 4 (5.8%)	0.744 0.461 0.567
Peritoneal irritation and Abdominal guarding	37 (9.1%)	15 (7.1%)	11 (8.7%)	11 (15.9%)	0.027

A total of 233 patients (57.2%) underwent laparoscopic appendectomy. The use of this minimally invasive approach decreased progressively with advancing age. Specifically, 132 patients (62.6%) in group G1, 74 patients (58.3%) in group G2, and only 27 patients (39.1%) in group G3 received laparoscopic surgery. This age-related difference in surgical approach was statistically significant (p = 0.003).

Histopathological findings of the excised appendices, stratified by age group, are presented in **Table 3**.

**Table 3:** Morphopathological type.

Morphopathological examination	All, n=407	G1, n=211	G2, n=127	G3, n=69	p
Catarrhal appendicitis	78 (19.2%)	57 (27%)	19 (15%)	2 (2.9%)	<0.001
Phlegmonous appendicitis	219 (53.8%)	120 (56.9%)	66 (52%)	33 (47.8%)	
Gangrenous appendicitis	110 (27%)	34 (16.1%)	42 (33%)	34 (49.3%)	

The incidence of generalized peritonitis also varied significantly across age groups. It was observed in 9 patients (4.3%) in G1, 16 patients (12.6%) in G2, and 17 patients (24.6%) in G3 (p < 0.001).

Postoperative complications were rare, with surgical site infections reported in 4 cases (0.98%). Of these, 3 cases (0.73%) occurred in G2, and 1 case (0.24%) in G1.

Three postoperative deaths were recorded, all occurring in G3 patients.

The mean length of hospital stay was 5.27 ± 1.73 days for G1 patients, increasing to 6.46 ± 3.15 days for G2, and reaching 8.52 ± 4.46 days for the oldest group. A significantly longer hospitalization duration was observed with increasing age (p < 0.001).

The variation of blood cell count and inflammatory markers across the different age groups are presented in **Table 4**.

**Table 4:** Variation of inflammatory markers depending on age group.

Marker	All, n=407	G1, n=211	G2, n=127	G3, n=69	p
Lymphocytes (*10^9/L)	1.947 ± 0.957	2.011 ± 0.945	2.039 ± 1.048	1.584 ± 0.717	0.002
Monocytes (*10^9/L)	0.542 ± 0.524	0.470 ± 0.386	0.643 ± 0.700	0.574 ± 0.482	0.011
Platelets (*10^9/L)	258.983 ± 72.605	256.998 ± 68.562	265.837 ± 71.559	252.439 ± 85.589	0.394
Neutrophils (*10^9/L)	11.212 ± 4.469	11.147 ± 4.482	11.597 ± 4.350	10.708 ± 4.350	0.394
NLR	7.72 ± 6.73	7.6 ± 673	7.58 ± 5.39	8.35 ± 5.37	0.650
LMR	6.69 ± 8.56	6.94 ± 4.72	7.37 ± 13.74	4.7 ± 3.73	0.096
PLR	170.77 ± 132.09	163.37 ± 143.95	170.33 ± 117.81	194.18 ± 117.17	0.243
AISI	1040.08 ± 1350.48	866.43 ± 1086.56	1312.28 ± 1722.52	1070.09 ± 1229.84	0.013
SIRI	3.75 ± 2.67	3.07 ± 2.36	4.57 ± 3.45	4.28 ± 3.59	0.005
SII	1955.43 ± 1519.13	1889.61 ± 1518.17	1564.23 ± 2243.62	1584 ± 7171	0.652

Subsequently, we analyzed the variation of parameters according to the postoperative histopathological exam of the excised appendix.

The demographic characteristics are summarized in **Table 5**. Moreover, the symptoms and signs patients presented with at the time of admission, are presented in **Table 6**.

**Table 5:** Demographic characteristics.

Characteristic	All, n=407	HP1, n=78	HP2, n=219	HP3, n=110	p
Age, years (M ± SD)	38.94 ± 16.63	30.58 ± 10.59	38.19 ± 16.21	46.36 ± 17.86	<0.001
Gender, men	243 (59.7%)	33 (42.3%)	142 (64.8%)	68 (61.8%)	0.002
Urban	216 (53.1%)	39 (50%)	113 (51.6%)	64 (58.2%)	0.440

**Table 6:** Signs and symptoms depending on HP type.

Signs and symptoms	All, n=407	HP1, n=78	HP2, n=219	HP3, n=110	p
Symptoms					
Nausea	238 (59.7%)	39 (50%)	128 (58.4%)	71 (64.5%)	0.137
Vomiting	149 (36.6%)	24 (30.8%)	80 (36.5%)	45 (40.9%)	0.364
Signs					
Fever	133 (32.7%)	18 (23.1%)	68 (31.1%)	47 (42.7%)	0.014
Peritoneal irritation	57 (14%)	7 (9%)	41 (18.7%)	9 (8.2%)	0.021
Abdominal guarding	28 (6.9%)	9 (11.5%)	14 (6.4%)	5 (4.5%)	>0.362
Peritoneal irritation and Abdominal guarding	37 (9.1%)	4 (5.1%)	19 (8.7%)	14 (12.7%)	0.027

An association was observed between gangrenous appendicitis and the presence of nausea at admission (p = 0.048) as well as fever (p = 0.004).

Regarding the type of surgical intervention, 233 patients (57.2%) underwent laparoscopic appendectomy. Among those with catarrhal appendicitis (HP1), 44 patients (56.4%) were treated using the laparoscopic approach. In the phlegmonous group (HP2), 138 patients (63.0%) underwent minimally invasive surgery, while in the gangrenous group (HP3), the proportion decreased to 51 patients (46.4%). These differences were statistically significant (p = 0.016).

Generalized peritonitis was observed in a total of 42 patients. It occurred in only 1 patient (2.4%) in the HP1 group, in 10 patients (4.6%) in HP2, and in 31 patients (28.2%) in HP3. The distribution across groups was statistically significant (p < 0.001).

Postoperative complications, specifically surgical site infections, were reported in 4 cases (0.98%). These included 2 cases (0.49%) in HP1, 1 case (0.24%) in HP2, and 1 case (0.24%) in HP3.The mean length of hospital stay was 5.71 ± 2.67 days for HP1 patients, 5.56 ± 2.06 days for HP2, and 7.80 ± 4.23 days for HP3 patients. A significantly longer hospitalization was observed with increasing histopathological severity (p < 0.001).

All postoperative deaths occurred among patients in the HP3 group.

The variation of blood cell count and inflammatory markers across the different histopathological groups are presented in **Table 7**.

**Table 7:** Variation of inflammatory markers depending on HP type.

Marker	All, n=407	HP1, n=78	HP2, n=219	HP3, n=110	p
Lymphocytes (*10^9/L)	1.947 ± 0.957	2.432 ± 0.879	1.924 ± 0.967	1.650 ± 0.857	<0.001
Monocytes (*10^9/L)	0.542 ± 0.524	0.491 ± 0.770	0.541 ± 0.408	0.579 ± 0.517	0.530
Platelets (*10^9/L)	258.983 ± 72.605	250.709 ± 69.500	266.281 ± 72.543	250.321 ± 73.934	0.091
Neutrophils (*10^9/L)	11.212 ± 4.469	7.789 ± 3.558	11.703 ± 4.098	12.661 ± 4.568	<0.001
NLR	7.72 ± 6.73	4.28 ± 5.21	8.04 ± 6.28	9.53 ± 5.4	<0.001
LMR	6.69 ± 8.56	7.83 ± 3.96	5.89 ± 4.5	7.47 ± 14.78	0.123
PLR	170.77 ± 132.09	122.71 ± 72.82	176.33 ± 144.96	193.77 ± 130.24	0.001
AISI	1040.08 ± 1350.48	465.02 ± 754.44	1121.6 ± 1356.97	1285.565 ± 1548.42	<0.001
SIRI	3.75 ± 4.21	1.65 ± 2.21	3.9 ± 4.21	4.93 ± 5.25	<0.001
SII	1955.42 ± 1519.13	1047.58 ± 1039.85	2092.96 ± 1589.75	2325.35 ± 1418.27	<0.001

### 3.1 Multivariable multinomial logistic regression

Multivariable multinomial logistic regression analysis was performed to assess the independent associations between age, length of hospital stay, systemic inflammatory indices (e.g., Lymphocyte count, Monocyte count, Neutrophil count, Platelet count, NLR, LMR, PLR, SII, SIRI, AISI) and the histopathological subtypes of acute appendicitis.

All the results of the mutinominal regression are presented in **Table 8**.

**Table 8:** Multivariable multinominal regression.

Parameter Estimates									
Hp^a^		B	Std. Error	Wald	df	Sig.	Exp(B)	95% Confidence Interval for Exp(B)	
								Lower Bound	Upper Bound
HP2	Intercept	-4.371	1.524	8.220	1	.004			
	Age	.051	.013	14.471	1	.000	1.052	1.025	1.080
	Length of hospital stay	-.154	.079	3.804	1	.051	.857	.735	1.001
	Lymphocyte	.050	.309	.026	1	.872	1.051	.573	1.926
	Monocyte	-1.353	.788	2.952	1	.086	.258	.055	1.210
	Platelet	.002	.005	.199	1	.656	1.002	.993	1.011
	Neutrophil	.378	.108	12.181	1	.000	1.459	1.180	1.804
	NLR	-.079	.117	.449	1	.503	.924	.735	1.163
	LMR	-.021	.033	.393	1	.531	.980	.919	1.045
	PLR	.013	.008	2.545	1	.111	1.013	.997	1.030
	AISI	-.002	.001	3.899	1	.048	.998	.996	1.000
	SII	-.001	.001	1.663	1	.197	.999	.998	1.000
	SIRI	.961	.399	5.792	1	.016	2.615	1.195	5.720
HP3	Intercept	-7.466	1.750	18.204	1	.000			
	Age	.064	.015	19.191	1	.000	1.066	1.036	1.097
	Length of hospital stay	.120	.079	2.305	1	.129	1.128	.966	1.317
	Lymphocyte	-.469	.384	1.491	1	.222	.625	.294	1.328
	Monocyte	-.566	.558	1.029	1	.310	.568	.190	1.694
	Platelet	-.006	.006	.907	1	.341	.994	.983	1.006
	Neutrophil	.588	.123	23.036	1	.000	1.800	1.416	2.289
	NLR	-.258	.152	2.863	1	.091	.773	.573	1.042
	LMR	.046	.022	4.366	1	.037	1.047	1.003	1.093
	PLR	.020	.009	5.065	1	.024	1.020	1.003	1.037
	AISI	-.002	.001	5.075	1	.024	.998	.996	1.000
	SII	-.001	.001	1.449	1	.229	.999	.998	1.001
	SIRI	1.089	.405	7.214	1	.007	2.970	1.342	6.574

a. The reference category is: HP1.

Significant results obtained were the following:

HP2 vs. HP1:
  Age: OR = 1.052 (95% CI: 1.025–1.080, *p* < 0.001) → Increasing age was significantly associated with a higher likelihood of phlegmonous appendicitis.
  Neutrophil Count: OR = 1.459 (95% CI: 1.180–1.804, p < 0.001) → Elevated neutrophil levels independently predicted phlegmonous histology.
  AISI: OR = 0.998 (95% CI: 0.996–1.000, p = 0.048) → Lower AISI values were significantly associated with phlegmonous appendicitis.
  SIRI: OR = 2.615 (95% CI: 1.195–5.720, p = 0.016) → Higher SIRI scores were independently correlated with an increased risk of phlegmonous forms.
HP3 vs. HP1:
  Age: OR = 1.066 (95% CI: 1.036–1.097, p < 0.001) → Advancing age remained a strong predictor of gangrenous transformation.
  Neutrophil Count: OR = 1.800 (95% CI: 1.416–2.289, p < 0.001) → Neutrophilia demonstrated a robust association with gangrenous pathology.
  LMR: OR = 1.047 (95% CI: 1.003–1.093, p = 0.037) → An elevated LMR was significantly linked to gangrenous appendicitis.
  PLR: OR = 1.020 (95% CI: 1.003–1.037, p = 0.024) → Higher PLR values were independently associated with more severe histological forms.
  SIRI: OR = 2.970 (95% CI: 1.342–6.574, p = 0.007) → SIRI was a strong and independent predictor of gangrenous appendicitis.

## 4 Discussions

Acute appendicitis (AA) remains one of the most common causes of abdominal surgical emergencies, and its clinical spectrum—from simple catarrhal inflammation to gangrenous necrosis—requires early and accurate severity stratification. Our retrospective study of 407 adult patients offers valuable insights into how systemic inflammatory indices, derived from routine hematologic parameters, may function as adjunctive tools in preoperative risk assessment. The observed associations between PLR, SII, SIRI, and histopathological severity support the growing emphasis on immunologically informed diagnostics, particularly in high-risk and elderly populations.

NLR is one of the most widely studied inflammatory markers in AA. Elevated NLR reflects systemic inflammation through neutrophil predominance and lymphocyte suppression, which are commonly associated with advanced histopathological stages. In our cohort, increased neutrophil counts were significantly correlated with gangrenous appendicitis. These findings are consistent with prior evidence indicating that NLR is significantly higher in complicated versus uncomplicated AA (3, 7, 21). Duyan M et all (22), confirmed NLR’s high specificity and sensitivity in predicting complicated AA, especially in male and elderly cohorts. Similarly, Karatas T et al. demonstrated that NLR had a superior discriminative ability compared to CRP alone (23).

PLR while less studied, has garnered attention for its dual involvement in thrombocyte response and lymphocyte suppression. This combined hematologic dynamic reflects both acute-phase reactant activation and immune modulation, making PLR a potentially useful surrogate for systemic inflammation in acute appendicitis. In our analysis, PLR showed moderate predictive value for gangrenous forms, consistent with data from literature, where PLR values were significantly higher in patients with perforated appendicitis (7, 24, 25). However, findings across studies remain heterogeneous; a recent evaluation failed to show PLR as an independent predictor when adjusted for age and comorbidities (10). This divergence reinforces the importance of contextual interpretation, particularly in elderly patients, where thrombocyte dynamics may be influenced by vascular pathologies or medication use.

A transient decrease in circulating monocytes observed in our study—particularly among patients with phlegmonous appendicitis—may reflect peripheral recruitment of these cells into the inflamed appendiceal tissue. Monocytes serve as precursors for macrophages and dendritic cells, which are essential for antigen presentation, tissue remodeling, and the resolution of inflammation. This proposed mechanism aligns with previous reports where monocyte depletion was noted in acute inflammatory conditions, including gastrointestinal and systemic infections (7, 10). Furthermore, studies by Uludağ SS et al. (25) and Zarog M. et al. (26) underscore the dynamic role of monocytes in modulating immune responses during acute surgical inflammation.

An important contribution of our study is the use of multivariable multinomial logistic regression instead of binary logistic modeling. This approach allowed for nuanced differentiation across histological subtypes—catarrhal, phlegmonous, and gangrenous—rather than simplistic binary groupings. While most previous studies limited themselves to binary endpoints (e.g., complicated vs. uncomplicated), our stratification showed that markers such as SIRI and neutrophil count are more discriminative of phlegmonous inflammation, while PLR, LMR, SIRI and neutrophils are stronger predictors of gangrene (27). However, due to the lack of diagnostic performance analyses, it is important to acknowledge that ORs are measures of association rather than indicators of diagnostic accuracy. We believe it is important to clarify this distinction, in order to have a more nuanced interpretation of these findings and prevent potential overstatements.

Composite indices such as SII and SIRI, which incorporate neutrophils, lymphocytes, monocytes, and platelets, emerged as more reliable markers in our multivariable multinomial logistic model, especially for phlegmonous and gangrenous appendicitis. Cakcak İE et al. (10) highlighted that both SII and SIRI outperform CRP and NLR in early diagnosis and severity assessment of AA. Their superiority lies in integrating three cellular pathways—innate response (neutrophils, monocytes), adaptive suppression (lymphocytes), and thrombocytic activation—providing a multi-dimensional immune snapshot (10, 28). SIRI did achieve statistical significance for both phlegmonous (*p* = 0.016) and gangrenous (*p* = 0.007) appendicitis, further validating its potential as a robust inflammatory marker. Given SIRI’s integrative nature—capturing both innate activation and adaptive suppression—its role in severe histopathological forms remains biologically plausible and merits further investigation in larger prospective studies.

Age played a significant role as an independent risk factor for gangrenous appendicitis. We acknowledge, however, that this relationship is associative rather than causal, and that factors such as comorbidities or variations in disease presentation may also contribute to the observed biomarker patterns in older adults. This is in alignment with current understanding of immunosenescence, where aging is associated with chronic low-grade inflammation, increased neutrophilic tone, and decreased lymphocyte responsiveness. Other studies present similar age-related shifts in inflammatory parameters, with elevated NLR, PLR, and CRP in elderly patients presenting with complicated appendicitis (10, 21, 25, 28, 29). These findings underscore the need to integrate age as a covariate in predictive models, especially when using immune-derived indices.

Our data also align with clinical performance studies of simple laboratory markers in preoperative triage. studies demonstrated that NLR, SII, and monocyte counts had better discriminative ability than total white blood cells in predicting perforation risk (6, 29). Their findings emphasize the global applicability of these markers, even across resource-limited settings, and support the inclusion of SII and SIRI in rapid diagnostic algorithms.

One of the most practical implications of our work is the integration of these indices into existing clinical pathways. Numerous scoring systems (e.g., Alvarado, AIR, RIPASA) exist, yet many lack immunologic depth. The incorporation of biomarkers such as SII or SIRI could enhance predictive accuracy, particularly in equivocal cases. Recent proposals by Cakcak İE et al. (10) suggest that hybrid models, combining lab indices with clinical features, outperform traditional scores alone in reducing negative appendectomy rates.

Our study has several strengths. It includes a relatively large adult cohort, spans six years of data, and uses rigorous histopathological confirmation. Additionally, by excluding patients with COVID-19 or active malignancies, we controlled for confounding sources of systemic inflammation.

However, limitations must be acknowledged. The retrospective nature and single-center design restrict external validity. Firstly we took into consideration all patients who underwent surgery and met the inclusion criteria, so no prior sample size calculation was performed. Furthermore, we did not explore serial changes in biomarkers or integrate biochemical markers such as procalcitonin or IL-6. Moreover, the lack of pediatric and immunosuppressed cases limits the generalizability of our findings to broader populations.

Future research should explore prospective multicenter validation of our findings, ideally integrating cytokine panels, flow cytometry of immune subsets, and even AI-based modeling. Particularly, real-time calculation of indices such as SII and SIRI could be automated within hospital systems, enabling stratified triage in emergency settings. Given the growing emphasis on precision surgery, immunologically driven stratification may represent the next evolution in appendicitis management.

## 5 Conclusions

In conclusion, systemic inflammatory indices—especially NLR, PLR, SII, and SIRI—demonstrate considerable promise as adjunctive tools for preoperative stratification in acute appendicitis. When interpreted within a multivariable multinomial framework and adjusted for demographic variables such as age, these markers offer a nuanced, accessible, and clinically relevant dimension to surgical decision-making. Our findings contribute to the evolving evidence base that supports immunologic personalization in emergency surgical care, with potential to improve diagnostic accuracy, streamline care, and reduce unnecessary imaging or interventions.

## Data Availability

The original contributions presented in the study are included in the article/supplementary material. Further inquiries can be directed to the corresponding authors.
